# Supplementary material for: Landscape of allele-specific transcription factor binding in the human genome
Source: Nat Commun. 2021 May 12;12:2751. doi: 10.1038/s41467-021-23007-0 (PMC8115691; doi:10.1038/s41467-021-23007-0)
Supplement: Supplementary file 3 — Reporting Summary [file 41467_2021_23007_MOESM3_ESM.pdf]

nature research

Corresponding author(s): IVAN V KULAKOVSKIY, VSEVOLOD J MAKEEV

Last updated by author(s): 19 MARCH 2021

Reporting Summary

Nature Research wishes to improve the reproducibility of the work that we publish. This form provides structure for consistency and transparency in reporting. For further information on Nature Research policies, see our Editorial Policies and the Editorial Policy Checklist.

Statistics

For all statistical analyses, confirm that the following items are present in the figure legend, table legend, main text, or Methods section.

n/a	Confirmed	
☐	☒	The exact sample size (n) for each experimental group/condition, given as a discrete number and unit of measurement
☐	☒	A statement on whether measurements were taken from distinct samples or whether the same sample was measured repeatedly
☐	☒	The statistical test(s) used AND whether they are one- or two-sided<br>*Only common tests should be described solely by name; describe more complex techniques in the Methods section.*
☒	☐	A description of all covariates tested
☐	☒	A description of any assumptions or corrections, such as tests of normality and adjustment for multiple comparisons
☒	☐	A full description of the statistical parameters including central tendency (e.g. means) or other basic estimates (e.g. regression coefficient) AND variation (e.g. standard deviation) or associated estimates of uncertainty (e.g. confidence intervals)
☐	☒	For null hypothesis testing, the test statistic (e.g. F, t, r) with confidence intervals, effect sizes, degrees of freedom and P value noted<br>*Give P values as exact values whenever suitable.*
☐	☒	For Bayesian analysis, information on the choice of priors and Markov chain Monte Carlo settings
☐	☒	For hierarchical and complex designs, identification of the appropriate level for tests and full reporting of outcomes
☐	☒	Estimates of effect sizes (e.g. Cohen's d, Pearson's r), indicating how they were calculated

Our web collection on statistics for biologists contains articles on many of the points above.

Software and code

Policy information about availability of computer code

Data collection	The analyzed ChIP-Seq data was extracted from the GTRD database: http://gtrd.biouml.org.
Data analysis	The following public software was utilized during data analysis:<br>bowtie 2, https://github.com/BenLangmead/bowtie2<br>PICARD 2.18.25, https://broadinstitute.github.io/picard/<br>GATK 4.0.12.0, https://gatk.broadinstitute.org/hc/en-us<br>ChIPseeker 1.22, https://bioconductor.org/packages/release/bioc/html/ChIPseeker.html<br>scikit-learn 0.22.2, https://scikit-learn.org<br><br>The ADASTRA pipeline code is available at GitHub: https://github.com/autosome-ru/ADASTRA-pipeline, doi:10.5281/zenodo.4008546<br>BABACHI segmentation software is available at GitHub: https://github.com/autosome-ru/BABACHI, doi:10.5281/ZENODO.4008544<br>The custom code for machine learning analysis is available at GitHub:<br>https://github.com/autosome-ru/ASB-ML, doi:10.5281/ZENODO.4043865<br>The SPRY-SARUS motif scanner v2.0.2 is available at GitHub: https://github.com/autosome-ru/sarus

For manuscripts utilizing custom algorithms or software that are central to the research but not yet described in published literature, software must be made available to editors and reviewers. We strongly encourage code deposition in a community repository (e.g. GitHub). See the Nature Research guidelines for submitting code & software for further information.

Data

Policy information about availability of data

All manuscripts must include a data availability statement. This statement should provide the following information, where applicable:

- Accession codes, unique identifiers, or web links for publicly available datasets
- A list of figures that have associated raw data
- A description of any restrictions on data availability

The complete data on ASBs across TFs and cell types described in this study are available in the release 1.6.10-Soos of the ADASTRA database (http://adastra.autosome.ru/) and provided online: https://adastra.autosome.ru/soos/
The BAD maps generated in this study are available at https://adastra.autosome.ru/soos/downloads
The list of utilized GTRD datasets is available online: https://adastra.autosome.ru/assets/exps/ADASTRA_GTRD_exps.soos.tsv
The reprocessed ChIP-Seq peaks and metadata are available in the GTRD database: http://gtrd.biouml.org
The MCF7 DNA sequencing data used for constructing an independent BAD map is available in SRA: accession SRR8652105, https://www.ncbi.nlm.nih.gov/sra/?term=SRR8652105
The transcription factor binding motifs are available in the HOCOMOCO collection: https://hocomoco.autosome.ru

Field-specific reporting

Please select the one below that is the best fit for your research. If you are not sure, read the appropriate sections before making your selection.

☒ Life sciences ☐ Behavioural & social sciences ☐ Ecological, evolutionary & environmental sciences

For a reference copy of the document with all sections, see nature.com/documents/nr-reporting-summary-flat.pdf

Life sciences study design

All studies must disclose on these points even when the disclosure is negative.

Sample size	Our study is based on the 7669 ChIP-Seq read alignments from GTRD which provides the largest available source of reprocessed ChIP-Seq data.
Data exclusions	By design of the pipeline, ChIP-Seq datasets were excluded if not eligible for assessing the background allelic dosage, see the Methods for details.
Replication	By design of the pipeline, each ChIP-Seq read alignment was considered as independent experiment when performing the data aggregation.
Randomization	We performed deterministic analysis using GTRD read alignments so no randomization was possible by design.
Blinding	No blinding was performed as our study did not ivolve individual subjects.

Reporting for specific materials, systems and methods

We require information from authors about some types of materials, experimental systems and methods used in many studies. Here, indicate whether each material, system or method listed is relevant to your study. If you are not sure if a list item applies to your research, read the appropriate section before selecting a response.

Materials & experimental systems

n/a	Involved in the study	
☒	☐	Antibodies
☒	☐	Eukaryotic cell lines
☒	☐	Palaeontology and archaeology
☒	☐	Animals and other organisms
☒	☐	Human research participants
☒	☐	Clinical data
☒	☐	Dual use research of concern

Methods

n/a	Involved in the study	
☒	☐	ChIP-seq
☒	☐	Flow cytometry
☒	☐	MRI-based neuroimaging
